# Anesthesia-induced Developmental Neurotoxicity in Pediatric Population

**DOI:** 10.26502/jsr.10020400

**Published:** 2024-11-21

**Authors:** Fihr Chaudhary, Devendra K. Agrawal

**Affiliations:** Department of Translational Research, College of Osteopathic Medicine of the Pacific, Western University of Health Sciences, Pomona CA 91766, USA

**Keywords:** Anesthesia-induced developmental neurotoxicity, Apoptotic pathways, Congenital heart disease, Endoplasmic reticulum, GABA agonist, Hypoxia, Lysosomes, Mitochondrial dysfunction, Neuroapoptosis, Neurocognitive impairment, Neurodevelopmental outcomes, Neurotoxicity, Neurogenesis, NMDA antagonist, Pediatric anesthesia, Synaptogenesis

## Abstract

Anesthetics and sedatives may cause long-term negative neurocognitive consequences in children. Many clinical reports on this subject have had a profound impact on the field of clinical pediatric anesthesiology. Findings from animal models suggest that early exposure to anesthesia might cause neurocognitive impairment and apoptotic cell death in the brain. Even though the findings from the experimental animals cannot be directly translated to the use of anesthesia in pediatric population due to many variable factors, parents and government regulatory bodies have become sensitive and attentive to the potential adverse effects of anesthesia in children. Multiple epidemiological investigations in human have added to the growing body of evidence showing neurological impairment and cognitive decline after early anesthetic exposure. This is supported by several outcome indicators, including validated neuropsychologic testing, educational interventions for neurodevelopmental or behavioral disorders, and academic performance or school readiness. These outcomes have been evaluated in clinical studies involving children who have been subjected to general anesthesia. The primary goal of this article is to critically review the clinical findings, perform systematic analyses of the evidence, discuss potential underlying mechanisms of neurotoxicity, the pathophysiology of anesthesia-induced developmental neurotoxicity involving mitochondria, endoplasmic reticulum, and lysosomes, and the ethical considerations of pediatric anesthesia. Although detailed well-controlled clinical studies are warranted, the evidence so far support that the potential adverse effects of surgical anesthesia to induce neurotoxicity in pediatric population are not exaggerated.

## Introduction

There is a serious conundrum for healthcare providers: administering the needed analgesia in children could cause hyperalgesia, altered pain processing, chronic pain syndromes, and behavioral issues [1, 2], but the administration of too much anesthetic agent can have the opposite effect. Unfortunately, there are no known safe alternatives to the currently used anesthetics, and there are often urgent diagnostic tests or surgical procedures in children that require anesthesia, and these procedures cannot be put off. The complexity of the problem is increased by a few confounding elements. Some pediatric groups may be particularly susceptible to anesthetic neurotoxicity due to patient comorbidities and other environmental, biological, and social variables.

The effects of anesthetics on the developing brain have been investigated in both animal studies and human therapeutic trials. In this article, we present the findings of the systematic analyses of the experimental and clinical studies and discussed potential underlying mechanisms of neurotoxicity with the pathophysiology of anesthesia-induced developmental neurotoxicity.

## Anesthesia in Newborns

Surgical procedures and imaging examinations sometimes require general anesthesia on both full-term and preterm infants. Due to significant improvements in monitoring and the development of volatile anesthetics with less cardiac depressive effects, critically sick newborns can now safely undergo anesthesia. Experimental evidence suggests that nearly all anesthetics increase neuroapoptosis in developing animal models. Additionally, postoperative hypotension and hypocapnia can significantly impact the neurocognitive development of infants. It has been proven that anesthesia is crucial in lowering the risk of complications during surgery and the stress reaction that patients experience during the surgery [3]. When administered to neonates in conjunction with nitrous gas and a paralytic, the combination of fentanyl and these anesthetics enhanced postoperative outcome in a randomized trial [4] by reducing the risk of metabolic and circulatory problems. Similarly, another study [5] found that newborns who were anesthetized during gastroschisis repair were less likely to experience intestinal ischemia, complete parenteral feeding needs, and unanticipated reoperations than those infants who were not. While there has been considerable improvement in the safety of pediatric anesthesia over the past sixty years, with a current perioperative mortality rate of 0.9/10,000 anesthetics, the mortality rate for neonates is disproportionately greater compared to other age groups [6]. Infants younger than one year old have a mortality rate that is six-times that of neonates, while children younger than eighteen years old have a mortality rate that is twenty-five times greater. According to another study [7], the newborn physiology, perioperative co-morbidities, and the fivefold higher frequency of congenital heart disease in neonates are the main causes of their 69-fold higher mortality rate during anesthesia compared to children older than 10 years old. Adverse perioperative outcomes in neonates are most commonly caused by delays in administering anesthesia, which puts the youngest newborns at the greatest risk. In addition to hypoxia, hypotension, and hypoglycemia, these conditions are linked to negative neurocognitive effects in this age group.

## Animal Studies of Anesthesia-associated Neurotoxicity

Animal studies have shown that newborns exposed to anesthesia have permanent impairment in learning and memory. Among the many types of anesthetics, two groups are known to mainly cause cell death in neurons: those that act as N-methyl-D-aspartate (NMDA) antagonists (such as nitrous oxide and ketamine) and those that act as gamma-amino butyric acid (GABA) agonists (such as midazolam, propofol, and volatile anesthetics). In 1999, a seminal study described extensive neuroapoptosis in newborn rats exposed to midazolam and nitrous oxide [8]. Later research has found that most general anesthetics, such as benzodiazepines, nitrous oxide, isoflurane, sevoflurane, thiopental, propofol, and ketamine, hasten cell death in developing rats, nematodes, pigs, and primates [9]. Despite neuroapoptosis being a natural component of human development, studies on animals have shown that maladaptive apoptotic patterns can cause developmental delays or even total paralysis [10]. Exposure to NMDA and GABA drugs for longer periods of time had the most profound effects on neonatal primates and rats younger than 7 days [11]. Animals not only died, but also showed changes in dendritic spines and astroglial growth, adverse effects on neurogenesis, decreased synaptic density, mitochondrial degeneration, and diminished neurotrophic factors [12]. Some have shown disturbing changes in behavior and learning capacity over extended periods of time [13]. Due to many variable factors, these results in animals may not be directly applicable to people. When studying complex operations in human neonates, researchers rely on continuous hemodynamic monitoring and repeated assessments of acid-base status and glucose levels. However, these measures are not always available in animal models. While under anesthesia, rats are at significant risk for developing metabolic acidosis, hypoglycemia, and hypercarbia, all of which can damage their neurological systems. These abnormalities would require immediate attention and prompt treatment in a clinical setting.

To apply findings from animal studies to real-world situations, researchers need to know the pharmacokinetics and pharmacodynamics, of anesthetic agents in pediatric subjects. These required parameters include half-life of the anesthetic agents, effective dose for clinical benefit, the dose range that can induce neurotoxicity or toxicity to any other organ, such as kidney, liver, in children. It should be noted that animal models have employed quite high dosages of anesthetic drugs per kilogram of body weight and somewhat longer exposure times. Given that human life expectancy is substantially higher than that of the experimental animal, extrapolation the findings on the effects of anesthesia duration to human presents several questions and challenges. Research on animals has shown that substances including dexmedetomidine, xenon, melatonin, and β-estradiol have a protective effect on the nervous system [14].

## Human Trials of Anesthesia-related Neurotoxicity

Brain development in humans is at its peak between the ages of 28 weeks gestation and 2 years of age. Research involving cohorts of people has mostly focused on youngsters less than four years old. There is conflicting evidence from these human trials; some reveal negative effects on neurodevelopment in children who have general anesthesia, while others find no correlation at all, even when looking at experiments involving identical twins or siblings.

Debilitating neurologic effects were initially associated with general anesthesia in one research [15]. They asserted that young children exposed to vinyl ether, ethyl chloride, and cycloprone for otolaryngologic surgery exhibited night terrors, bed wetting, tantrums, and frightened behaviors.

Neonates and infants having congenital heart surgery, whether for palliative or remedial purposes (e.g., hypoplastic left heart syndrome), are one group that is regularly monitored with neuropsychological testing. Evidence suggests that these newborns suffer from neurocognitive impairment because of circulatory arrest, abnormalities in regional cerebral perfusion, deep hypothermia, abnormal preoperative brain function, decreased perioperative oxygen and cardiac output, the use of cardiopulmonary bypass, and other factors [16]. Babies that had the newborn artery switch procedure showed impairments in cognitive and motor development in one study [17] that looked at standardized testing. Potentially caused by many exposures to anesthetics, these cognitive impairments may persist over time.

Critically sick newborns with necrotizing enterocolitis [18] who had to have surgery while under anesthesia had worse neurodevelopmental outcomes and a growth retardation than babies who were medically treated. Due to their higher need for inotropic support and more severe illness than toddlers and children, babies born with an exceptionally low birth weight may have skewed these results. This article compared a group of children who were less than three years old and had hernia repairs done under general anesthesia to another group of children of the same age who were not exposed to the anesthetic agent. Pediatric hernia repair patients had a diagnosis of a developmental or behavioral abnormality that persisted after adjusting for gender and congenital illness [18].

Mayo Clinic researchers examined around 5,000 kids from birth to school age in 2009 and found that those who had two or more anesthetics were more likely to have learning impairments [19]. However, a different study [20] evaluated the pairs of identical twins from the Netherlands and discovered that the twins whether exposed to anesthesia or not managed similarly in terms of intellectual success. These conflicting results could be due to many confounding factors and require further attention.

Early Development Index (EDI) scores are a measure of a child’s preparedness for school. A population-based study in Canada [21] examined the correlation between surgical exposure in young children and the EDI scores. After adjusting for age, gestational age at birth, and socioeconomic characteristics, these investigators discovered a weak connection between early anesthetic exposure and low EDI score. There was no correlation between exposure to numerous anesthetics and an increased risk, although there was an obvious increase in risk for children subjected to anesthesia after the age of 2 [21]. As a result, the authors speculated that the contradictory results might be caused by undiscovered confounders.

In the Pediatric Anesthesia and Neurodevelopmental Assessment (PANDA) [22] study, 105 sets of siblings from four separate pediatric facilities were compared to a sample of 8-to-15 years old who had undergone hernia repairs before the age of 3. A battery of neurodevelopmental tests, with full scale IQ as the main endpoint, is administered to children who have had surgery and compared to their siblings who are less than 36 months apart. Researchers in this study were unable to discern any significant changes between children who had undergone anesthesia and those who had not in terms of motor skills, processing speed, language, visuospatial ability, attention, executive function, or behavioral skills.

One of the first randomized controlled trials to evaluate general anesthesia to regional anesthesia for inguinal hernia surgery in healthy newborns was the 2016 General Anesthesia Compared to Spinal Anesthesia (GAS) trial [23]. When comparing cognitive tests at age 2 after 80 minutes of exposure to sevoflurane anesthesia vs spinal anesthesia, no significant difference was seen. Anesthesia exposure before the age of five was examined in one 2017 article [24] involving 38,493 children who underwent common surgical procedures such as pyloromyotomy, inguinal hernia repair, tonsillectomy, and circumcision other than during the newborn period. Fifty confounders, including sociodemographic characteristics, medical comorbidities, and health care consumption, were used to propensity score match exposed children to five control groups. Specifically, the study found that developmental delay and attention-deficit hyperreactivity disorder (ADHD) had an increased risk, leading to an overall hazard ratio of 1.26 for pediatric mental illnesses.

It is critical to point out that one cannot just look at the negative consequences of anesthesia on learning, memory, and cognition. It is important to thoroughly investigate potential confounding factors that may be associated with perioperative risk factors, patient comorbidities, the type and duration of surgery, underlying disease, and environmental factors. Thus, to investigate the impact of anesthetic on the developmental outcomes in children, several epidemiological neurotoxicity studies have sought to account for socioeconomic variables, such as the educational status of the mother, and comorbidities, such as congenital heart disease.

## Mechanisms of Anesthetic-Induced Neurotoxicity

When a child is exposed to a general anesthetic before synaptogenesis has occurred in the brain, it can cause detrimental neurological effects known as anesthetic-induced developmental neurotoxicity (AIDN).

**Figure 1:**
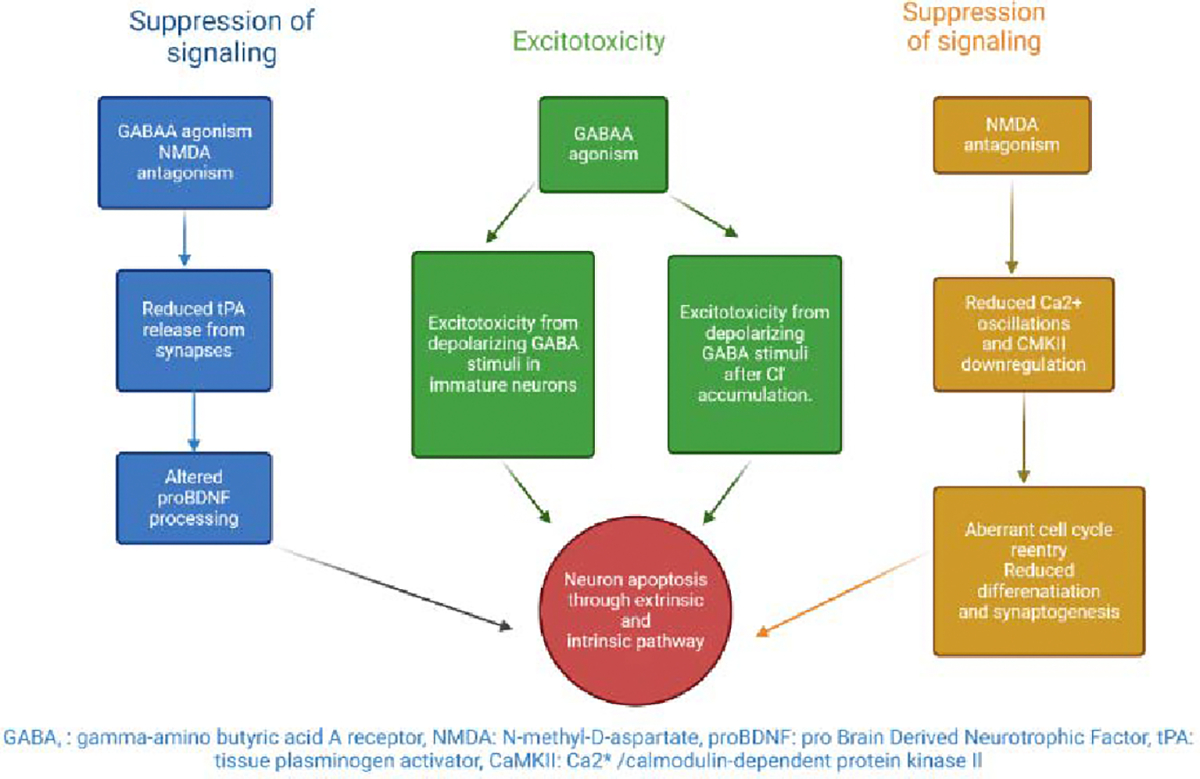
Potential underlying mechanisms of neurotoxicity involving suppression of signaling and excitotoxicity leading to neuronal apoptosis through intrinsic and extrinsic pathways. BDNF, brain-derived neurotrophic factor; GABAA, gamma-amino butyric acid receptor A which is an ionotropic receptor and ligand-gated ion channel; NMDA, N-methyl-D-aspartate is a glutamate receptor; tPA, tissue plasminogen activator is a serine protease.

Neuroapoptosis appears to be caused in part by the potentiation of GABAA receptors and the antagonism of NMDA receptors. It has been shown that the administration of a combination of anesthetics or intrauterine exposure to ethanol, which has both NMDA antagonistic and GABAA agonistic effects, causes widespread programmed cell death, suggesting that these mechanisms work together [9]. Some theories have been advanced to account for general toxicity and two primary ones for anesthesia-induced neurotoxicity (Figure 1).

The agents cause harm to the amygdala and hippocampus, which triggers problems with learning and memory. It has been discovered that both glutaminergic and GABAergic neurons may undergo apoptosis upon exposure to excessive glutamate. Reports suggest that anesthetics inhaled into the air can activate nicotinamide adenine dinucleotide phosphate (NADPH) oxidase. This can cause an excess of superoxide to be produced, as well as mitochondrial malfunction and cell death. Variations in intracellular calcium homeostasis and changes in the expression of ligand-gated ion channels are two more hypothesized pathways. Agent type, concentration, and length of exposure are other variables thought to contribute to AIDN [25]. Multiple anesthetic exposures in children may cause developmental problems in adulthood, even while brief exposures may be harmless.

## Studies addressing AIDN

Inhalational anesthetic exposure throughout childhood has been the subject of few prospective studies that have looked at the possibility of AIDN in children. Researchers in the Pediatric Anesthesia Neuro Development Assessment (PANDA) project [21] compared 105 sets of siblings, one with and one without, before the age of 36 months. The exposed siblings had inguinal hernia surgery while the unexposed siblings did not. The inhalational anesthetic was exposed to the subject for 20–240 minutes. Statistical analysis of neurocognitive and behavioral outcomes measured by IQ in later childhood using data on anesthetic exposure that had been previously recorded did not reveal any significant differences.

Another study comparing general anesthesia (GA) with awake-regional anesthesia (anesthetic gases – GAS) in infants randomized babies to get awake-regional anesthesia instead of GA for inguinal herniorrhaphy [26]. The risk of unfavorable neurodevelopmental outcome at 2 years of age was not higher with sevoflurane anesthesia for 1 hour compared to awake regional anesthesia, according to the authors. An ongoing MASK study at the Mayo Clinic is looking at how many anesthetics given to children before the age of three affect their neurocognitive abilities [27]. Children born in Sweden between 1973 and 1993 were the subjects of a cohort research [28]. The researchers looked at the correlation between having surgery or anesthesia before the age of four and cognitive and academic performance in the years following, using IQ test results from military conscription and grade point averages at the age of sixteen. A performance analysis was conducted on the students from April 2013 to October 2015. They discovered a weak population-level connection between exposure to anesthesia and surgery before the age of four and cognitive or academic performance in adolescents.

**Figure 2:**
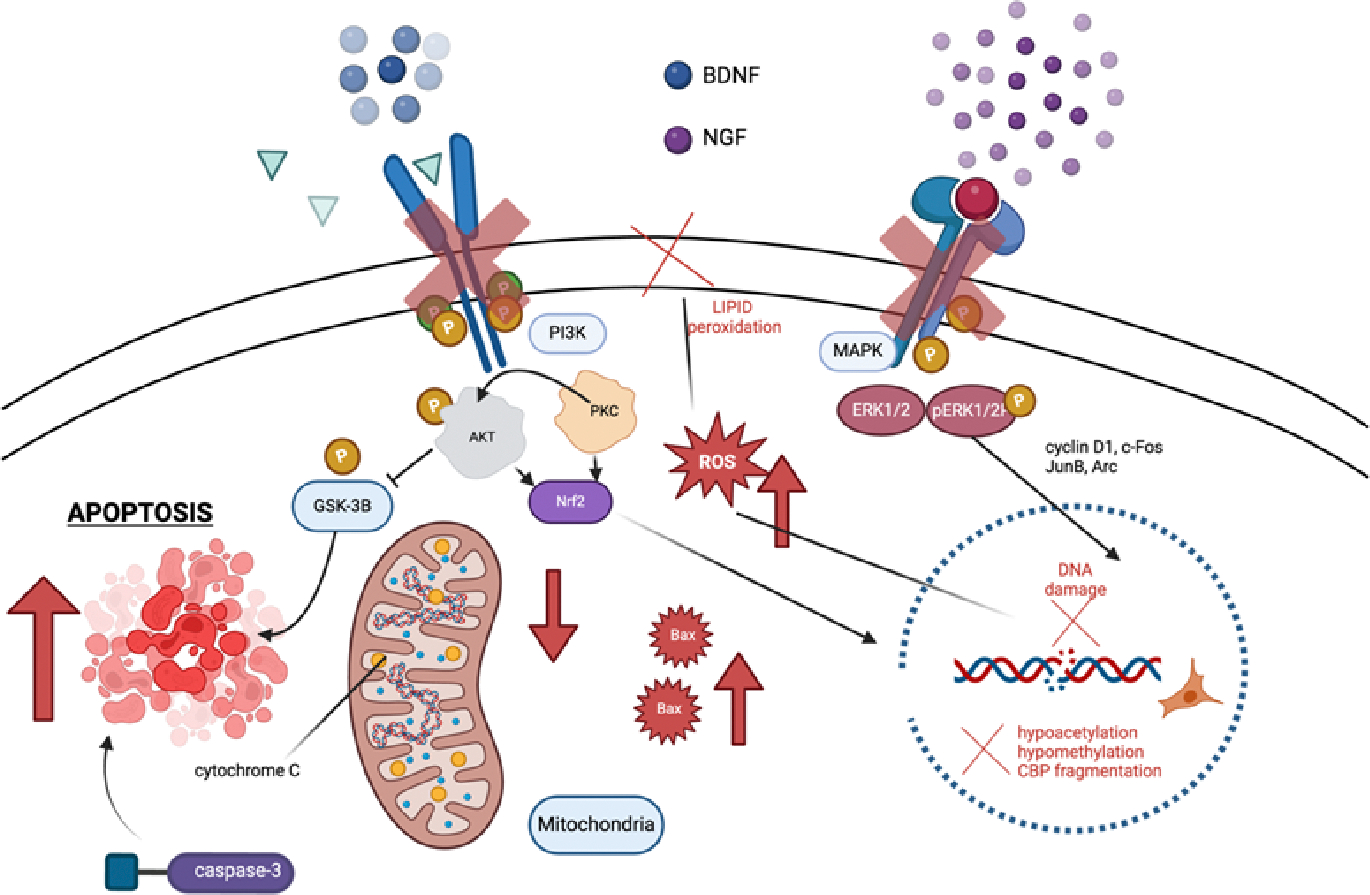
An overview of the key cellular targets in the pathophysiology of general anesthesia (GA)-induced developmental neurotoxicity. The key mechanisms by which GA cause harm to developing neurons are illustrated by red arrows and crosses. There are two main kinase routes that growth factors use to transduce survival signals, and GA prevents neurons from receiving these signals. Also, GA can cause cytochrome c exudation and cell death by directly inducing ROS production, genetic and epigenetic abnormalities, and mitochondrial instability. When neuroprotective techniques are activated, the black borders mark the locations at which neurotoxicity is reversed. BDNF, brain-derived neurotrophic factor; NGF, nerve growth factor; ROS, reactive oxygen species.

GA disrupts highly controlled signal transduction pathways, which are essential for neuronal survival and development in their early stages of development and have evolved to be very conserved [21]. Two main kinases, PI3K/Akt [25] and MAPK/ERK [26], are typically phosphorylated and activated by growth factors such neural growth factor (NGF) and brain-derived neurotrophic factor (BDNF). If left unchecked, phosphorylated Akt (pAkt) causes glycogen synthase kinase 3 beta (GSK-3β) to become inactive and eventually induce apoptosis by an unknown mechanism [26]. After being phosphorylated, ERK (pERK) moves into the nucleus, where it reduces the expression of cell cycle inhibitors (JunB, Arc) and promotes the transcription of survival-related genes (cyclin D1, c-Fos) [25]. The harmful consequences of GA are depicted in figure 2.

## Role of Mitochondria in Anesthesia-Induced Developmental Neurotoxicity

Early events activate an apoptotic cascade that is dependent on mitochondria [29], which implies that mitochondria could be a potential target. The activation of effector caspases is the final step in various metabolic processes that lead to apoptosis. As shown in Figure 2, the mitochondria-dependent pathway leads to apoptosis by decreasing levels of bcl-2 family anti-apoptotic proteins (e.g., bcl-xL), increasing permeability of the mitochondrial membrane, and finally, increasing cytochrome c release into the cytoplasm, which activates caspase-9 and caspase-3. A drop in bcl-xL protein levels, an increase in cytochrome c, and activation of caspase-9 are indicators that GA given during synaptogenesis initiate the mitochondria-dependent cascade within the first two hours following anesthetic exposure [29].

A mitochondria-dependent apoptotic cascade is activated as a very early event [29], indicating that mitochondria could be a susceptible target. There are various biochemical processes that lead to apoptosis and the activation of effector caspases. An increase in mitochondrial membrane permeability, a decrease in bcl-2 family anti-apoptotic proteins (such as bcl-xL), and an increase in cytochrome c release into the cytoplasmic Golgi apparatus also led to a persistent disruption of mitochondrial morphogenesis through the mitochondria-dependent pathway. After being exposed to anesthesia for two weeks, the mitochondria showed signs of enlargement together with damaged inner membranes and disorganized cristae, indicating a serious breakdown in the integrity of the mitochondrial membranes [30].

Understanding how anesthesia-induced morphological changes impact mitochondrial migration is equally important, as mitochondria must migrate within the cytoplasm to distribute within cells [31]. Previous research has concentrated on the morphological appearance of mitochondria located at the neuronal body, where they are generated.

The mitochondria, which are responsible for regulating ATP synthesis, are often located near active synapses [32] and growth cones [31] in growing neurons. According to previous research, there appears to be a considerable decrease in the number of mitochondria present in the presynaptic neuronal profiles of brain tissue that has been subjected to anesthesia compared to control subjects [33]. Anesthesia is thought to make mitochondria slow and “stuck” in more proximal cellular compartments, which would explain why they enlarge mitochondria even more. This would mean that mitochondria are no longer distributed regionally along very thin and highly arborized dendritic branches, which are essential for normal synapse formation and development. Others have found that anesthesia hinders synaptic development, stability, and function in addition to dendritic spine plasticity. The medial prefrontal cortex of P5 rats showed a decrease in synaptic spine density when propofol was administered, but rats aged P15–P25 showed an increase in spine density [11]. In the medial prefrontal cortex of P16 rats, one study [34] discovered that isoflurane, sevoflurane, or desflurane influenced synaptogenesis (increased dendritic spine density) but did not cause cell death.

**Figure 3:**
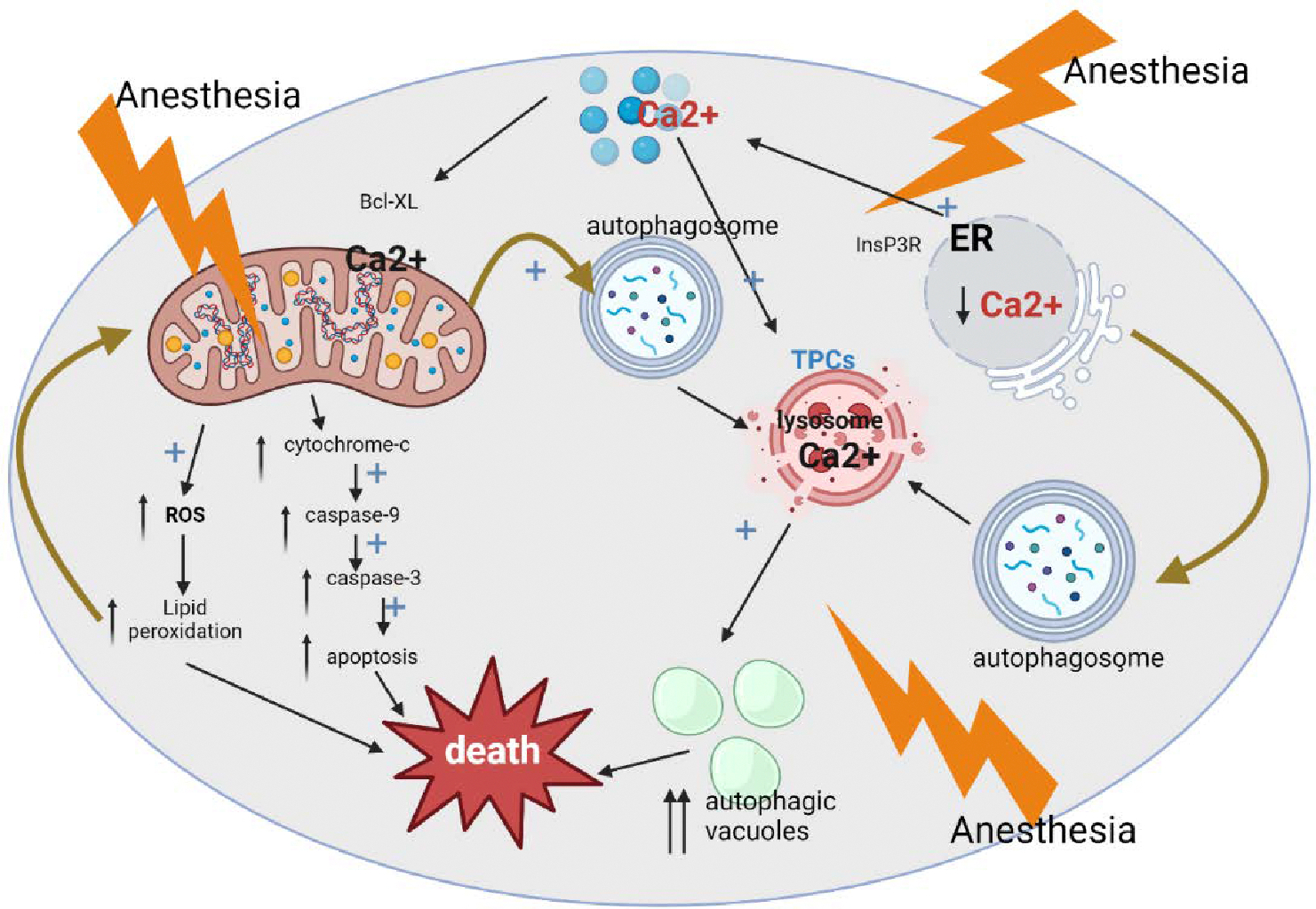
A schematic depicting the developmental neurodegenerative pathways brought about by anesthesia. Mitochondria, the endoplasmic reticula (ER), and lysosomes are the key components of three different pathways: (i) Anesthesia-induced activation of inositol 1,4,5-trisphosphate receptor (InsP3R) is part of the ER-dependent pathway that causes an abrupt increase of cytosolic Ca^2+^ and excessive calcium (Ca^2+^) release. The result is a decrease in the levels of bcl-xL, a protein that protects mitochondria from cell death, which triggers cytochrome c leakage in the cytoplasm. To trigger the mitochondrial apoptotic pathway, which in turn causes DNA fragmentation and neuronal death, cytochrome c must first activate caspase-9 and caspase-3. (ii) In the mitochondria-dependent pathway, anesthesia-induced ROS up-regulation damages neuronal organelles, mitochondria, and the endoplasmic reticulum (ER) in particular, and causes excessive lipid peroxidation of lipid membranes. Overproduction of autophagosomes and a rise in autophagic load result from the removal of damaged mitochondria, which can become an unmanageable source of reactive oxygen species (ROS) and cytochrome c, and damaged endoplasmic reticulum (ER), which can become an uncontrollable source of cytosolic calcium (Ca^2+^). (iii) A lysosome-dependent mechanism regulates Ca^2+^ uptake into lysosomes through nicotinic acid adenine dinucleotide phosphate (NAADP) gated two-pore channels (TPCs). When the intraliposomal Ca^2+^ level rises, lysosomal activity is activated, leading to the creation of autophagic vacuoles, lysosomal and autophagosome fusion, and neuronal “self-eating.” It is not yet known if anesthesia directly affects lysosomal activation (via NAADP-gated TPCs in particular), but it is thought that anesthesia indirectly causes it by increasing cytosolic Ca^2+^ from the ER.

When mitochondria are damaged, what happens? Excessive reactive oxygen species (ROS) production and substantial peroxidation of cellular and subcellular lipid membranes occur alongside morphological distortion and impairment of mitochondrial regional distribution [31]. This plays a significant role in the onset and course of several neurological illnesses characterized by a sharp inability to think clearly [35]. To synthesize ATP, neurons rely heavily on glucose, and mitochondrial oxidative phosphorylation results in the production of ROS. Neural cells are extremely vulnerable to an overabundance of ROS due to their high oxygen demand and relative lack of oxidative defenses, namely, low to moderate catalase and manganese superoxide dismutase (SOD) activity. Because of their weakness and the high concentration of polyunsaturated fatty acids in them, they are prone to cellular damage and severe lipid peroxidation [31]. It is true that developing neurons are more vulnerable to ROS up-regulation, mitochondria-induced ROS-propagated lipid peroxidation, and neuronal deletion when exposed to anesthesia at an early stage. These factors could potentially lead to the cognitive development impairment observed, as well as activate caspase-9 and caspase-3, causing apoptosis (Figure 3). An increase in cytochrome c, a decrease in bcl-xL protein levels, and activation of caspase-9 are all indicators that GAs given during synaptogenesis activate the mitochondria-dependent cascade within the first two hours following anesthetic exposure [29].

## Role of Endoplasmic Reticulum in Anesthesia-Induced Developmental Neurotoxicity

It is important to remember that the reported impairment of mitochondrial function and its downstream repercussions could have an upstream cause—excessive calcium release from the ER, leading to cytosolic and mitochondrial calcium overload. If this happens, it might lead to cytochrome c leak [36], which would exacerbate mitochondrial dysfunction (Figure 3). It is possible that the endothelium-rich zone (ER) is one of the primary sites of neurotoxicity caused by anesthesia. In fact, one study demonstrated conclusively [37] that the inhalational anesthetic isoflurane promotes apoptotic neuronal death in the developing brain of rats by activating inositol 1,4,5-trisphosphate receptors, which in turn causes a substantial release of calcium from the endoplasmic reticulum (ER), leading to an abrupt increase of cytosolic calcium and modulation of mitochondrial bcl-xL protein. There have been reports of similar effects with several GA, including propofol, desflurane, and sevoflurane on the inositol 1,4,5-trisphosphate receptors, leading to an increase in the activity of the mitochondrial permeability transition pore and cytosolic calcium excess [38]. The results showed that mitochondrial enlargement, caused by this enhanced pore activity, resulted to the uncontrolled release of pro-apoptotic proteins.

## Role of Lysosomes in Anesthesia-Induced Developmental Neurotoxicity

There is a real concern that anesthesia can lead to the production of numerous defective organelles, commonly known as toxic biological “garbage,” which must be broken down to protect neurons from harm. This is because mitochondrial damage could lead to an uncontrollable release of free oxygen radicals, and endoplasmic reticulum damage could cause intracellular calcium to remain uncontrolled. Through autophagy, large organelles can be removed without inflicting significant harm to neurons. The production of autophagosomes begins the multi-step process of autophagy [39]. Autophagosomes next enter lysosomes, an acidic vacuolar compartment. As a result of slow autophagocytization, defective organelles build up an auto fluorescent, polymeric substance called lipofuscin in their lysosomes.

One area of the brain that is particularly vulnerable to the neurotoxic effects of anesthesia is the developing subiculum, where pyramidal neurons have a high concentration of apoptotic markers such as autophagosomes, lysosomes, and autophagic vacuoles during anesthesia. Because of the increased autophagic burden, it is possible that anesthesia causes the death of developing neurons by causing “autophagic stress” - either by increasing lysosomal activity to dangerous levels or by overpowering natural autophagy because of an excess of faulty organelles. Previous models of cell damage have hinted at a possible connection between autophagy and neuroapoptosis. While some claim autophagy is necessary for apoptosis to begin [40], others maintain the two processes are separate [41]. One could argue that anesthesia-induced “self-eating” could be caused by reduced lysosome function, however it is unclear if GA have a role in activating this route. An essential aspect of endosomal-lysosomal trafficking is the regulation of lysosome calcium concentration by nicotinic acid dinucleotide phosphate (NAADP)-gated two-pore channels (TPCs) [42]. Even though most research has focused on diseases characterized by impaired autophagy rather than enhanced autophagy, it is not out of the question that GA cause a more direct disruption of lysosomal function when calcium levels are high, leading to an overactivation of endosomal-lysosomal trafficking, through mitochondrial damage, ROS up-regulation, and lipid peroxidation.

## Pharmacological Interventions for Preventing AIDN

Although some medicines have been tested and shown to lower AIDN, the exact mechanism by which they do so remains unclear. These include melatonin, lithium, dexmedetomidine, L-carnitine, 7-nitroindazole, and xenon [3]. Ciproxifan and Apocynin are two novel investigational medications that show promise in the treatment and reversal of AIDN. Ciproxifan, an H3-receptor antagonist with an imidazole moiety, causes an increase in the release of acetylcholine in the hippocampus, prefrontal cortex, and entorhinal cortex, as well as dopamine and norepinephrine in the prefrontal cortex [43]. Mice were exposed to isoflurane for 2 hours in an experimental trial, and then 24 hours later, they were given 1–3 mg/kg of ciproxifan intravenously. After 30 minutes of giving ciproxifan, they saw that the cognitive impairment went away.

*Picrorhiza kurroa* is a plant native to the Alpine-Himalayan region; its roots contain the biologically active compound apocynin, also known as acetovanillone or 4′-hydroxy-3′-methoxyacetophenone. Orally administered apocynin is a potent and selective inhibitor of phagocyte NADPH oxidase Nox2 [45]. Another study [6] exposed 6-day-old mice to neonatal sevoflurane 30 minutes before the procedure by injecting 50 mg/kg of apocynin into their peritoneum. The exposed mice were put through a contextual fear conditioning test when they were 11–13 weeks old. In addition to protecting basolateral amygdala glutamatergic neurons expressing c-Fos, they discovered that apocynin prevented learning deficits. Several mental problems, stroke, Alzheimer’s disease, Parkinson’s disease, and other neurological diseases have been documented to benefit from apocynin’s neuroprotective properties by other studies [7]. Researchers have administered apocynin to patients suffering from prostate cancer, nephrolithiasis, diabetic nephropathy, rheumatoid arthritis, and cardiovascular disorders due to its antioxidant characteristics [22]. The suggested way it works is by lowering superoxide levels and stopping additional mitochondrial dysfunction.

## Ethical Considerations in Pediatric Anesthesia

In the field of pediatric anesthesiology, the most important ethical concerns revolve around informed consent, informed permission, the best interest standard, informed assent, and the competence to make their own decisions. Anesthesiologists who specialize in pediatrics encounter several ethical challenges, including those pertaining to normal anesthetic care, the maturity and confidentiality of adolescents, informed refusal, pediatric research, and end-of-life care. Advocacy, citizenship, production pressure, the damaged parent, and refusal of transfusion therapy are some of the other topics that are related to ethics in pediatric anesthesiology.

## Conclusions

Neurotoxicity because of anesthesia in humans is a poorly understood clinical issue. When it comes to human neurotoxicity, there are prospective randomized placebo-controlled trials and retrospective research even though they are limited by confounders. The US Food and Drug Administration (FDA) issued a safety statement in 2016 warning that children under the age of three or pregnant women in the third trimester may have their brain development negatively impacted using general anesthesia for longer than three hours. Eleven sedatives and anesthetics, including benzodiazepines, are covered by this safety alert. An initiative to guarantee the safety of anesthesia for infants and children is the SmartTots (Strategies for Mitigating Anesthesia-Related Neurotoxicity in Tots), a public-private cooperation between the Food and Drug Administration and the International Anesthesia Research Society.

Every patient contact is a unique opportunity for practitioners to assess the risks and advantages of administering anesthesia, especially when it comes to time-sensitive treatments like surgery, imaging, or the insertion of a pacemaker in unborn children. There must be further study on the neurotoxicity of anesthesia, and the available facts do surely warrant a shift in pediatric anesthesia. In a coordinated effort in reaction to the FDA warning, the American Academy of Pediatrics reassured practitioners and parents that most of the research do not link a single brief exposure to anesthesia with any developmental difficulties. Based on the human trials covered in this analysis, there are inconsistent study outcomes.

Despite the retrospective character of the studies, many separate investigations have found some links between anesthesia and maladaptive cognitive outcomes in otherwise healthy children less than 2 years old, even when exposed to multiple injections of the drug. Additional research is needed to confirm these findings, but these studies indicate that healthy newborns and children exposed to a single anesthesia pose some degree of damage to their neurodevelopment in the long run. It is possible that the cognitive capacity of children is better determined by their biological, social, and environmental circumstances. Research in the future might aim to identify which groups and procedures pose the biggest danger to developing brains while under anesthesia.

## Key points

- Animal studies show that early exposure to anesthetics may cause neurocognitive impairments and apoptotic cell death in the brain.
- Anesthesia is necessary in pediatric surgery, but concerns exist about neurodevelopmental outcomes due to anesthesia exposure, especially in neonates.
- Studies indicate NMDA antagonists (e.g., ketamine) and GABA agonists (e.g., propofol) are implicated in causing neuro-apoptosis in developing animals.
- There is conflicting evidence from human trials; some show cognitive decline in children exposed to anesthesia, while others find no significant difference.
- Risk factors like hypoxia, hypotension, and preoperative conditions impact neonatal neurodevelopment under anesthesia.
- Mitochondrial dysfunction and oxidative stress are implicated in anesthesia-induced neurotoxicity through apoptotic mechanisms.
- Socioeconomic factors, comorbidities, and environmental factors may confound studies on the neurotoxicity due to pediatric anesthesia.
- Neuroprotective agents like dexmedetomidine, xenon, and melatonin have shown some protective effects against anesthesia-induced neurotoxicity.
- The PANDA and GAS trials show limited-to-no long-term cognitive impact of anesthesia but stress the importance of further research.
- Early brain development between 28 weeks’gestation and 2 years of age is critical, making this a high-risk period for anesthesia-induced neurodevelopmental effects.
